# Design and Optimization of Hierarchical Porous Metamaterial Lattices Inspired by the Pistol Shrimp’s Claw: Coupling for Superior Crashworthiness

**DOI:** 10.3390/biomimetics10090582

**Published:** 2025-09-02

**Authors:** Jiahong Wen, Na Wu, Pei Tian, Xinlin Li, Shucai Xu, Jiafeng Song

**Affiliations:** 1 School of Automotive Engineering, Shandong Jiaotong University, Jinan 250357, China; 2 Suzhou Automobile Research Institute (Xiangcheng), Tsinghua University, Suzhou 215133, China; 3 Centre for Composite Materials and Structures, School of Astronautics, Harbin Institute of Technology, Harbin 150080, China; 4 State Key Laboratory of Intelligent Green Vehicles and Mobility, Tsinghua University, Beijing 100084, China

**Keywords:** bionic design, mechanical metamaterials, negative Poisson’s ratio, crashworthiness, multi-objective optimization

## Abstract

This study, inspired by the impact resistance of the pistol shrimp’s predatory claw, investigates the design and optimization of bionic energy absorption structures. Four types of bionic hierarchical porous metamaterial lattice structures with a negative Poisson’s ratio were developed based on the microstructure of the pistol shrimp’s fixed claw. These structures were validated through finite element models and quasi-static compression tests. Results showed that each structure exhibited distinct advantages and shortcomings in specific evaluation indices. To address these limitations, four new bionic structures were designed by coupling the characteristics of the original structures. The coupled structures demonstrated a superior balance across various performance indicators, with the EOS (Eight pillars Orthogonal with Side connectors on square frame) structure showing the most promising results. To further enhance the EOS structure, a parametric study was conducted on the distance d from the edge line to the curve vertex and the length-to-width ratio y of the negative Poisson’s ratio structure beam. A fifth-order polynomial surrogate model was constructed to predict the Specific Energy Absorption (SEA), Crush Force Efficiency (CFE), and Undulation of Load-Carrying fluctuation (ULC) of the EOS structure. A multi-objective genetic algorithm was employed to optimize these three key performance indicators, achieving improvements of 1.98% in SEA, 2.42% in CFE, and 2.05% in ULC. This study provides a theoretical basis for the development of high-performance biomimetic energy absorption structures and demonstrates the effectiveness of coupling design with optimization algorithms to enhance structural performance.

## 1. Introduction

Mechanical metamaterials, as artificially engineered structural materials, possess mechanical properties that transcend those of traditional materials, primarily attributed to their customizable topological configurations [1,2]. The design of unit cell structures enables the realization of a range of counterintuitive mechanical properties, including Negative Poisson’s Ratio (NPR) [3,4], negative stiffness [5,6], negative compressibility [7], ultralow mass density [8], and high specific stiffness [9,10], among others.

Among the diverse array of metamaterial systems, NPR materials have garnered considerable attention due to their distinctive deformation coupling effects. In contrast to conventional materials, NPR materials exhibit lateral expansion under axial tension and lateral contraction under compression. This unconventional deformation behavior confers upon them a multitude of superior properties, including the optimization of energy absorption pathways through geometric reconstruction, progressive failure modes based on the synergy of microstructural buckling, and dynamic mechanical responses regulated by multiscale porosity [11,12,13]. In recent years, NPR materials have demonstrated significant application potential in various fields, including aerospace impact-resistant structures [14], vehicular traffic [15], impact protection [16], and biomedicine [17].

From the perspective of structural design, two-dimensional NPR configurations have been theoretically studied extensively. Classic configurations, such as re-entrant honeycombs, chiral lattices, and their composite variants (e.g., star-arrow hybrid unit cells), have been widely characterized and optimized [18,19,20,21,22]. Zhang et al. [23] constructed re-entrant double-arrow honeycomb (RDAH) structures to investigate the influence of impact direction and geometric parameters on dynamic platform stress.

Wang et al. [24] developed a star-arrow heterogeneous structure that exhibits a double-platform stress response under quasi-static compression, showing a significant enhancement in energy absorption efficiency per unit volume compared to traditional star honeycombs. Yu et al. [25], aiming to investigate the quasi-static compression performance and failure behavior of composite corrugated honeycomb sandwich structures, first calculated the relative density of the sandwich structure’s unit cells and fabricated three types of composite expandable honeycomb structures using carbon/epoxy resin: uniform expandable honeycomb, unidirectionally graded expandable honeycomb, and bidirectionally graded expandable honeycomb. These were utilized to compare the effects of gradient forms on mechanical properties. The distinctions among the three types reside in the stacking order, angles, and number of layers.

However, existing two-dimensional structures encounter limitations in practical engineering applications, including insufficient out-of-plane load-bearing capacity, pronounced anisotropy, and challenges in achieving coordinated control of the three-dimensional stress field [26,27,28].

In recent years, while some preliminary progress has been made in the design and fabrication of three-dimensional NPR structures, several key scientific challenges remain. Liu et al. [29] developed a three-dimensional helical lattice structure by integrating chiral rotation mechanisms with bio-inspired re-entrant topology, which achieved a 30% increase in specific energy absorption compared to traditional configurations and demonstrated its multidirectional impact resistance through helmet protection. Peng Wang et al. [30] extended the two-dimensional deep-sea glass sponge structure to three dimensions and further optimized the topology to design a new type of lattice called the modified body-centered cubic (MBCC) lattice. The results indicate that compared to traditional body-centered cubic designs and bio-inspired designs, the MBCC design can further enhance energy absorption capacity. The new lattice made of steel exhibits specific energy absorption close to that of foam made of titanium alloy. Wang et al. [31] proposed a novel microlattice composed of aperiodic unit cells inspired by Einstein’s tile (where cell orientations never repeat). Fabricated via 3D printing with high-strength rigid polymers, this aperiodic microlattice exhibits stable and progressive compressive deformation (in contrast to catastrophic fracture of periodic structures). At the same relative density, it outperforms periodic microlattices in key metrics: fracture strain (≥830% higher), energy absorption (≥300% higher), crush stress efficiency (≥130% higher), and smoothness coefficient (≥160% higher), showing excellent energy absorption and damage tolerance (retaining 76% ultimate stress after 30% compressive strain recovery).

Current research predominantly focuses on the mechanical responses of simple geometric topologies, leaving significant gaps in areas such as biomimetic structural optimization, adaptability to non-uniform dynamic loads, and mechanisms of multi-physical field coupling. Consequently, researchers have increasingly turned their attention to the sophisticated structures optimized by biological evolution, particularly those biological models that exhibit exceptional energy conversion efficiency. Biomimetic design has attracted widespread attention due to its capacity to draw inspiration from natural structures and functions [32,33]. For instance, Sun et al. [34], guided by biomimetic design principles, derived inspiration from the microstructure of pomelo peel to create a biomimetic lattice structure (y structure) that exhibits a negative Poisson’s ratio effect. To simplify the manufacturing process, they integrated the Y structure with a BCC structure, yielding four cohesive lattice structures that incorporate the Y structure. Cui et al. [35], inspired by the cuttlefish, devised a novel segmented bionic structure. The CL-H (cuttlebone-like hierarchical structure) lattice demonstrates exceptional mechanical properties with specific energy absorption values reaching up to tenfold those of non-hierarchical cuttlebone-like structures (CL) and triply periodic minimal surface structures.

The Pistol Shrimp has attracted significant attention owing to the unique closing mechanism of its predatory claw: the shock waves generated by the collapse of transient cavitation bubbles can reach a sound pressure level of 210 dB, while the spiral fiber-reinforced structure of the claw joint maintains excellent mechanical integrity under repeated impacts [36]. This bio-inspired energy absorption mode provides key insights into the development of novel impact-resistant metamaterials. However, traditional energy absorption structures continue to face challenges in weight, space, and energy absorption efficiency. The development of biomimetic and NPR-based structures offers promising solutions to these challenges. By integrating the unique properties of NPR materials with innovative designs inspired by natural organisms (such as Pistol Shrimp), advanced energy absorption structures with superior performance can be developed.

This paper integrates the biomimetic characteristics of the Pistol Shrimp’s claw, extracts the micro-characteristic curve of the Pistol Shrimp’s fixed claw, and designs a bionic energy absorption structure. Through experimental testing and finite element simulation, the crashworthiness and deformation processes of four different bionic Pistol Shrimp’s claw structures and four combined structures were investigated, and one structure with superior energy absorption performance was selected for multi-objective algorithm parameter optimization.

The content of this paper is organized as follows: Section 2 presents the design concept, parameter configuration, tensile testing of materials, and quasi-static compression testing of the model for the bionic Pistol Shrimp’s claw energy absorption structure. Section 3 details the establishment of the finite element simulation model and crashworthiness indicators for the bionic Pistol Shrimp’s claw energy absorption structure. Section 4 analyzes the crashworthiness and deformation processes of four different bionic Pistol Shrimp’s claw structures and four combined structures, parameterizes the EOS structure, and performs multi-objective optimization of its parameters. Section 5 compares the structure with others and showcases some potential application scenarios for the bionic Pistol Shrimp’s claw energy absorption structure. Section 6 concludes and provides future directions.

## 2. Bionic Design and Methods

This section presents the design concept and structural configuration of the bionic Pistol Shrimp’s claw negative Poisson’s ratio energy absorption structure and characterizes its material mechanical properties through raw material testing. In bionic design and biomechanical research, the precise extraction of biological morphological features holds significant importance. Analysis of biological morphological curves provides essential geometric insights for the design of bionic mechanical metamaterials, thereby optimizing their performance.

### 2.1. Structural Features of Pistol Shrimp

The morphologies of numerous flora and fauna in nature offer crucial inspiration for designing energy absorption structures that emphasize durability and lightweight characteristics. Notably, the Pistol Shrimp’s claw features a negative Poisson’s ratio energy absorption structure and an internal architecture constituted by a multicellular lattice, both of which hold substantial significance for the advancement of energy absorption mechanical metamaterials.

The Pistol Shrimp, formally known as Alpheus heterochaelis, derives its name from its ability to emit a high-speed water jet from its claw to stun prey. Adults can reach a maximum body length of 8 to 10 cm and are predominantly found in tropical marine environments.

**Figure 1:**
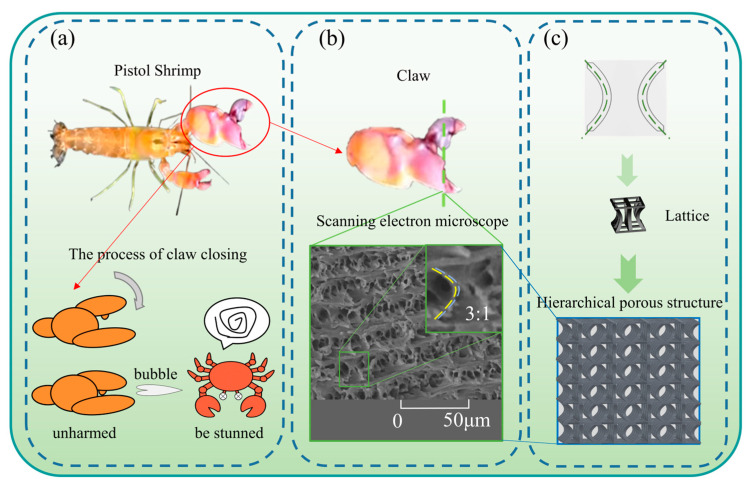
The design of a hierarchical porous metamaterial lattice structure with negative Poisson’s ratio inspired by the microscopic cross-sectional characteristics of the Pistol shrimp’s fixed claws: (**a**) the process by which the Pistol shrimps’ claws close to produce a high speed water column; (**b**) the claws of the Pistol shrimp and an electron microscope image of the fixed claws; (**c**) a hierarchical porous lattice structure design method based on the microscopic biological characteristics of the clawed lobster’s cut-off section.

The Pistol Shrimp’s claws vary in size, with the larger claw utilized for predation and the smaller one for feeding. As shown in Figure 1a, during predation, the outer claw of the larger claw rapidly closes, and the cylindrical protrusion swiftly inserts into the cavity of the fixed claw, ejecting a jet of water at approximately 30 m/s. This jet impacts the surrounding seawater and claw walls, forming bubbles that rapidly burst, producing a loud explosion and shock waves. The Pistol Shrimp employs this method to stun its prey and obtain food without causing self-injury [37]. Electron microscopy observations revealed that the dactyl of the shrimp claw exhibits a negative Poisson’s ratio structure of single unit cells at the microscopic level, with these cell arrays showing obvious stratification. The microscopic negative Poisson’s ratio structure has a buffering effect against impact loads. One of the unit cells was selected to extract the main features for the study.

The geometric contour characteristic curve structure of the bionic negative Poisson’s ratio multicellular structure inspired by the Pistol Shrimp’s claw is depicted in Figure 1b. This curve is a quadratic function curve fitted from a partial structure diagram extracted from Figure 1b through plotting scattered points.

(1) $$f(x)=-0.005988x^{2}+1.205x+4.747$$

**Figure 2:**
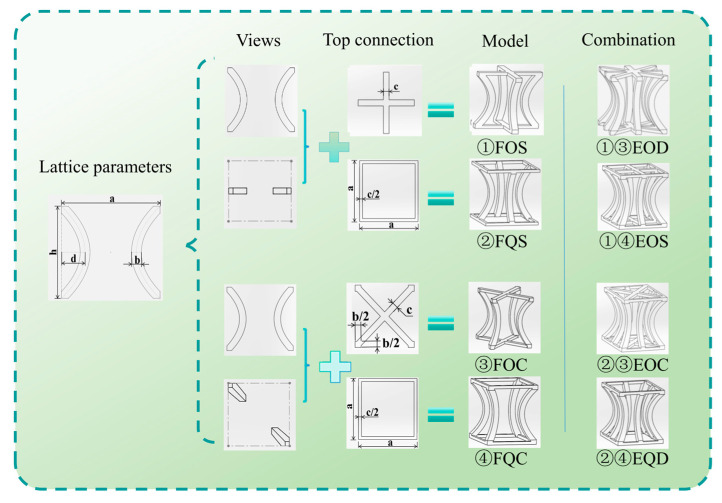
Dimensions of four bionic hierarchical porous metamaterial Lattice structures with a negative Poisson’s ratio of the Pistol shrimp’s claw and the combination process of the four combined structures (Number ①②③④ represents a structure; ①③, ①④, ②③, and ②④ represent new structures).

Inspired by this, corresponding bionic structures were designed in the study of negative Poisson’s ratio energy absorption structures, and their energy absorption and platform stress were investigated. Based on the configuration contour of the Pistol Shrimp’s claw, this paper proposes a bionic negative Poisson’s ratio multicellular structure inspired by the Pistol Shrimp’s claw. The simplified contour of the structure is depicted in Figure 2, with a design size of side length (*a*), overall height (*h*), equal distance lines on both sides of the curve with a distance of between the two lines (*b*), and a distance of from the curve vertex to the outer contour line (*d*).

### 2.2. Structural Design

Based on this structure, four types of F structures shown in Figure 2 were extended: (Four pillars Orthogonal with Side connectors, FOS), (Four pillars Quadrilateral with Side connectors, FQS), (Four pillars Orthogonal with Cross connectors, FOC), (Four pillars Quadrilateral with Cross connectors, FQC). The width of the orthogonal connections at the top and bottom ends is *c*, the width of the square contour is *c*/2 due to its unit cell array characteristics, and the same treatment method is used for the four corners of the diagonal orthogonal structure, which is *b*/2.

The study also investigates whether the four new structures formed by combining the four types of structures—(Eight pillars Orthogonal with Diagonal connectors, EOD), (Eight pillars Orthogonal with Side connectors on Square Frame, EOS), (Eight pillars Orthogonal with Cross connectors on square frame, EOC), and (Eight pillars Quadrilateral with Dual connectors, EQD)—can exhibit superior energy absorption characteristics. The combination process is illustrated with numbers in the figure. For instance, the EOD structure is a combination of FOS and FOC. The values of the parameters are presented in Table 1.

### 2.3. Material Tensile Test

**Figure 3:**
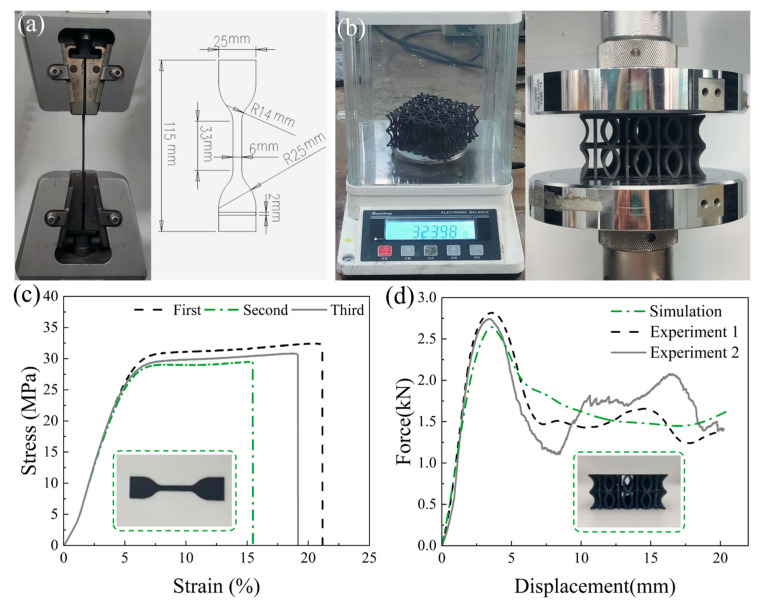
Material tensile test and sample compression test verification: (**a**) images of the tensile test site for class PA11 materials and the size of the tensile specimens; (**b**) images of the physical mass and quasi-static compression test site for the EOD model; (**c**) engineering stress–strain curves for the tensile testing of class PA11 materials; (**d**) force–displacement curves for the quasi-static compression test and simulation of the EOD model.

A universal testing machine was employed to perform quasi-static tensile tests on the tensile specimens of the matrix material. During the tests, to determine the Poisson’s ratio, axial and transverse strain gauges were attached to the gauge section of the PA11 tensile specimens: the axial gauge measured the strain along the loading direction, while the transverse gauge recorded the strain perpendicular to the loading direction. Figure 3a illustrates the tensile testing site and the dimensions of the tensile specimen for the class PA11 material. Figure 3c depicts the stress–strain curve of the class PA11 material under quasi-static uniaxial tension with a strain rate of 0.01 s^−1^, with the Poisson’s ratio calculated as the absolute value of the ratio of transverse strain to axial strain in the elastic deformation stage (prior to yielding), based on data from three repeated valid experiments. An ideal elastoplastic constitutive model was utilized to simplify the constitutive behavior in the simulation. The elastic modulus is 0.83 GPa, the yield strength is 27.14 MPa, the density is 990 kg·m^−3^, and the Poisson’s ratio is 0.36.

### 2.4. Quasi-Static Compression Test Verification

To verify the accuracy of the finite element model, specimens of the EOD structure were fabricated using Selective Laser Sintering (SLS) for experimental validation. This technology enables the production of complex-shaped structures with high dimensional accuracy without the need to consider internal support for the sample structure. As depicted in Figure 3b, the mass of the EOD specimen was initially measured, yielding a value of approximately 32.40 g. A universal testing machine, with a compression stroke set at 20 mm and a loading rate of 2 mm/min, was employed to perform quasi-static compression tests on the compression specimens. The quasi-static compression load–displacement curve of the EOD structure specimen is illustrated in Figure 3d.

The finite element model was configured with a compression amount of 50%, consistent with the quasi-static compression test, and the load–displacement curve of the EOD structure specimen simulated by the finite element model is depicted in Figure 3d. The comparison of the calculated index data between the quasi-static compression test and the finite element simulation is presented in Table 2. The error of Specific Energy Absorption (SEA) is 1.877% and −1.809%, the error of Peak Force (PF) is −4.618% and −2.043%, the error of Crush Force Efficiency (CFE) is −3.271% and 4.851%, the error of EA is 1.488% and −2.175%, and the error of m is 2.685% and 2.653%. The overall error does not exceed 5%, which is within the acceptable range, thus validating the finite element model’s capability to effectively simulate real working conditions.

## 3. Results

### 3.1. Finite Element (FE) Models

**Figure 4:**
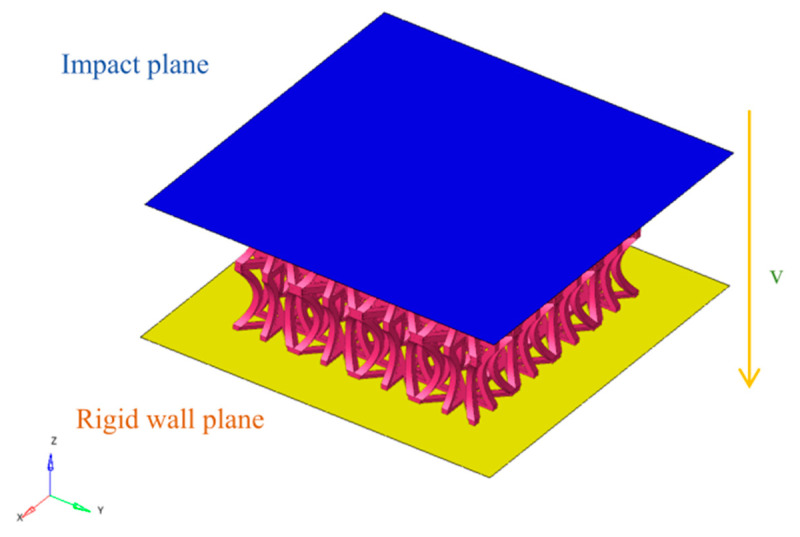
Finite element model.

A finite element model was developed using HYPERMESH/LS-DYNA, as depicted in Figure 4. The model comprises 4 unit cells in the X direction, 4 unit cells in the Y direction, and 2 unit cells in the Z direction, with an overall size of 80 mm × 80 mm × 40 mm. The geometric dimensions of the unit cells are detailed in Table 1.

The bottom rigid plate is fully constrained in all degrees of freedom, while the top rigid plate is constrained in all degrees of freedom except for movement along the Z-axis. The top rigid plate moves downward at a constant speed to compress the bionic structure. The bionic Pistol Shrimp’s claw negative Poisson’s ratio structure is in contact with the upper and lower rigid plates through surface-to-surface contact, with a friction coefficient set at 0.2. Additionally, self-contact is employed to prevent the negative Poisson’s ratio lattice structure from penetrating during compression, with the friction coefficient defined as 0.2.

Material failure due to fracture is not considered in the simulation process. The quasi-static compression of the EOD structure is simulated using the finite element analysis software LS-DYNA. To ensure quasi-static loading conditions, the kinetic energy must remain significantly lower than the internal energy throughout the loading process. To save computational time, the loading speed of the top rigid plate is set to 1 m/s, which satisfies the energy requirement [38].

### 3.2. Mesh Sensitivity

In the development of the finite element model for the EOD structure, mesh size exerts a substantial influence on both material properties and crashworthiness. Consequently, convergence analysis is essential for identifying a mesh size that balances high computational efficiency with accuracy.

**Figure 5:**
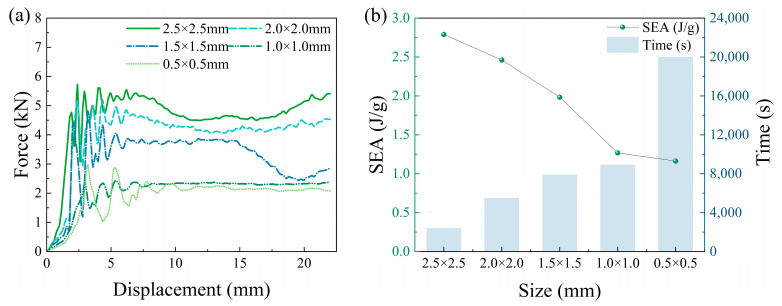
Mesh sensitivity analysis: (**a**) force–displacement curves for different mesh sizes; (**b**) computational time and specific energy absorption for different mesh sizes.

To determine the optimal mesh size for the model under collision conditions, the loading speed of the top rigid plate was set to 10 m/s. Convergence analysis was conducted using five mesh sizes: 0.5 mm × 0.5 mm, 1 mm × 1 mm, 1.5 mm × 1.5 mm, 2.0 mm × 2.0 mm, and 2.5 mm × 2.5 mm. As shown in Figure 5a, the load–displacement curves for the 0.5 mm × 0.5 mm and 1 mm × 1 mm meshes exhibit high similarity. Figure 5b demonstrates that the 1 mm × 1 mm mesh size yields relatively shorter computation time and higher accuracy.

### 3.3. Crashworthiness Indicators

Crashworthiness indicators are crucial for assessing energy absorption in structures subjected to impact. Commonly used indicators include Specific energy absorption (SEA), Initial peak force (IPF), Mean crush force (MCF), Crush force efficiency (CFE), and Undulation of Load-carrying fluctuation (ULC) [39].

EA signifies the energy absorbed during the crushing process, calculated as

(2) $$EA=\int_{a}^{b}f\left(x\right)dx$$

where *f*(*x*) is the function describing how the crushing force varies with displacement, with a and b marking the start and end points for energy absorption calculation.

SEA denotes the capacity to absorb energy per unit mass, as

(3) $$SEA=\frac{EA}{m}$$

where *m* represents the mass of the structure.

MCF indicates the average reaction force of the structure over the entire process, defined as the ratio of EA to the compression distance:

(4) $$MCF=\frac{EA}{b-a}$$

CFE serves as a metric for assessing the stability of energy absorption during the crushing process, computed by

(5) $$CFE=\frac{MCF}{PF}\times 100\%$$

PF here stands for the maximum load within the interval.

To achieve superior protective outcomes, effective energy absorption structures should maximize energy uptake during crushing while maintaining a stable absorption process.

ULC quantifies the fluctuation in the compression force–displacement curve. A smaller ULC implies a smoother curve and a more stable compression force, enhancing the structure’s energy absorption stability. It is formulated as

(6) $$ULC=\frac{1}{EA}\int_{a}^{b}\left|f\left(x\right)\right.-\left.MCF\right|\;dx$$

## 4. Results and Discussion

This chapter examines the crashworthiness and deformation processes of four different bionic Pistol Shrimp’s claw structures and four combined structures. Finite element simulation models for these bionic structures were developed based on the material mechanical properties and crashworthiness indicators obtained in Section 3. In order to improve the accuracy of energy absorption analysis, the total compression in the simulation was set to 60%.

### 4.1. The Crashworthiness and Deformation Process of Four Types of Bionic Structures (Group F)

**Figure 6:**
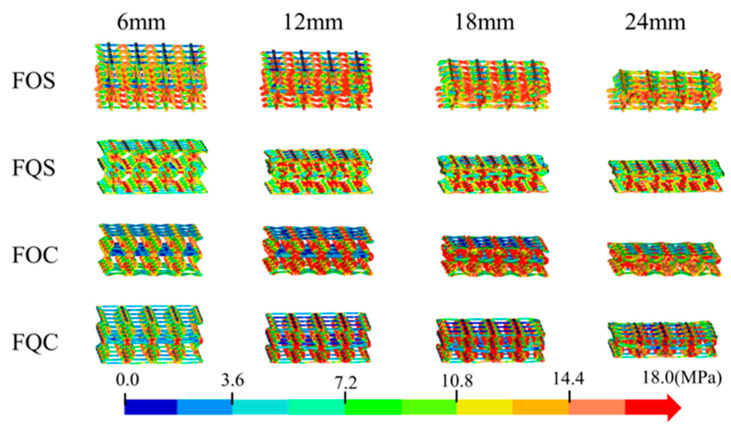
Stress cloud map of four types of bionic structures (Group F).

By comparing the stress distribution diagrams of different structures under varying compression strokes, the stress shifts and deformations of the bionic Pistol Shrimp’s claw negative Poisson’s ratio energy absorption structure were analyzed. Figure 6 presents the stress contour maps of the four bionic structures. The figure illustrates the deformation of the FOS, FQS, FOC, and FQC structures at compression strokes of 6 mm, 12 mm, 18 mm, and 24 mm. The color bar indicates the stress level, ranging from 0.0 MPa (blue) to 18.0 MPa (red).

At a compression stroke of 24 mm, the stress concentration area markedly expands, with stress levels ranging from 7.2 MPa to 18.0 MPa, indicating that the structure is more susceptible to local failure under high compression stroke.

The FQS structure, similar to the FOS structure, exhibits a smaller stress concentration area, indicating superior stress distribution uniformity under the same compression stroke. At a 24 mm compression stroke, the stress concentration area ranges from 3.6 MPa to 14.4 MPa, demonstrating good resistance to stress concentration.

The FOC structure demonstrates relatively uniform stress distribution and smaller stress concentration areas throughout all compression strokes, indicating good resistance to stress concentration. At a compression stroke of 24 mm, the stress distribution ranges from 7.2 MPa to 10.8 MPa, indicating that the structure maintains good stress distribution uniformity even under high compression strokes.

Under high compression strokes, the FOS and FOC structures are more susceptible to local failure, with stress concentration areas gradually expanding. The FQS structure demonstrates relatively uniform stress distribution throughout all compression strokes, indicating good resistance to stress concentration.

The FQC structure demonstrates relatively uniform stress distribution at a 6 mm compression stroke, but stress concentration begins to emerge at a 12 mm stroke, with the stress concentration area progressively expanding as the stroke increases.

**Figure 7:**
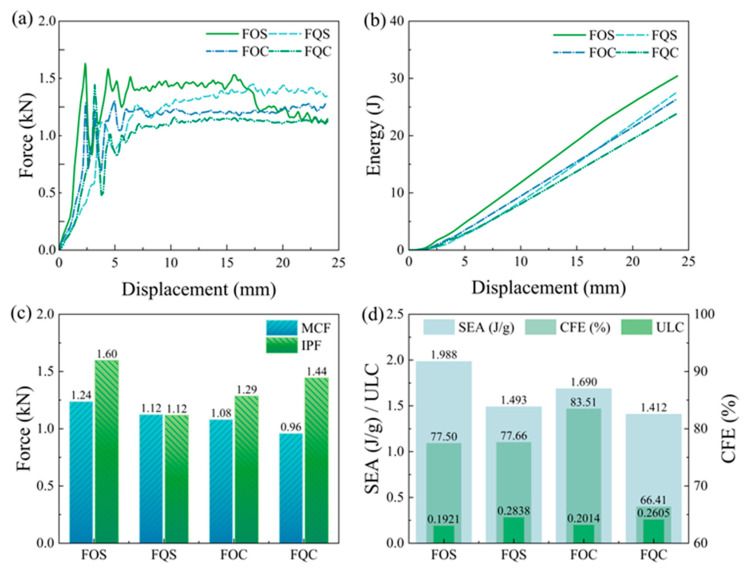
The crashworthiness indexes of the four types of bionic structures (Group F): (**a**) force–displacement curves; (**b**) energy absorption curves; (**c**) IPF and MCF; (**d**) SEA, CFE, and ULC.

To explore the energy absorption characteristics of the designed bionic Pistol Shrimp’s claw negative Poisson’s ratio energy absorption structure, Figure 7a presents the load–displacement curves of the four bionic structures.

At the onset of compression, the structure undergoes plastic strain, resulting in the initial peak force. As compression progresses, the curve exhibits regular fluctuations until the compression reaches 15 mm, When the platform stress of the FOS structure begins to decline. In conjunction with Figure 6, it is evident that this phenomenon is attributed to the poor overall stability of the FOS structure, leading to a tilt and displacement in the middle position. The load–displacement curves of FQS, FOC, and FQC show a general trend of consistency. Notably, the curve of FQS exhibits an upward trend compared to FOC and FQC. This observation is also corroborated by the energy absorption displacement curve in Figure 7b.

Figure 7c compares the MCF and IPF of the four structures, with FOS having the highest MCF and IPF. Notably, FQS has nearly identical MCF and IPF values, while FQC has the lowest MCF but a relatively high IPF. Figure 7d illustrates the three key crashworthiness indicators of the four structures: SEA, CFE, and ULC. Among them, the FOS structure exhibits the highest SEA of 1.988 J/g and the lowest ULC of 0.1921. The FOC structure demonstrates the highest CFE of 83.51%. The FQS structure has the lowest IPF of 1.2 kN and a relatively high CFE of 77.66%.

### 4.2. The Crashworthiness and Deformation Process of Four Types of Combination Structures (Group E)

In Section 4.1, it was observed that the performance indicators of the four bionic configurations exhibit singularity, limiting their widespread application in engineering. To address this limitation and to develop a bionic energy absorption structure with superior energy absorption and balanced performance, four combined structures, as depicted in Figure 2, were designed based on the structural characteristics of the four bionic structures.

**Figure 8:**
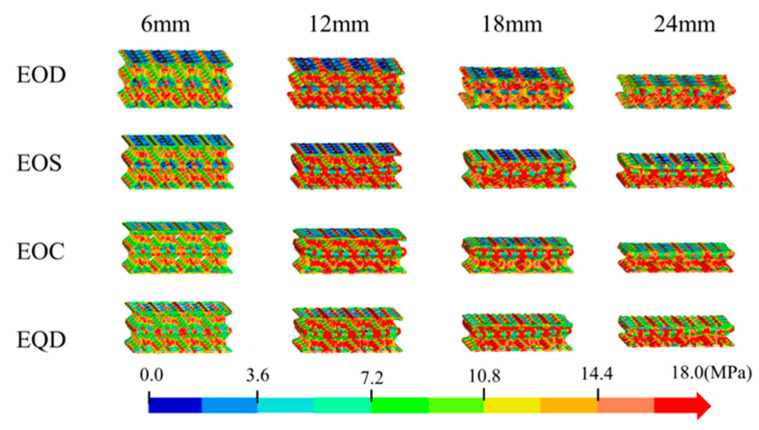
Stress cloud map of four types of combination structures (Group E).

As illustrated in Figure 8, the EOD configuration effectively disperses stress during the initial compression stage. However, slight displacement still occurs at the center position when the compression stroke is large. The EOS configuration demonstrates good stress dispersion capability, with lower stress concentration compared to EOD. At a compression stroke of 18 mm, the stress distribution further concentrates towards 10.8 MPa, yet maintains good stress dispersion. From the side view, EOS exhibits an inward concave curvature at the middle layer position, which is conducive to stress dispersion at the center. At a 24 mm stroke, the stress distribution reaches 14.4 MPa. Although stress concentration is observed, the stress distribution is more uniform compared to EOD. The EOC configuration shows excellent stress dispersion capability, with no obvious stress concentration. Compared to other configurations, the stress distribution is the most uniform. The EQD configuration exhibits a certain trend of stress concentration at a compression stroke of 12 mm. At a compression stroke of 18 mm, obvious stress concentration occurs. From the side view, the middle layer connection position of EQD differs from that of EOS, maintaining a roughly straight line that tilts to the left. This may be attributed to the unique configuration of EQD, distinct from EOD, EOS, and EOC, leading to differences in internal deformation.

**Figure 9:**
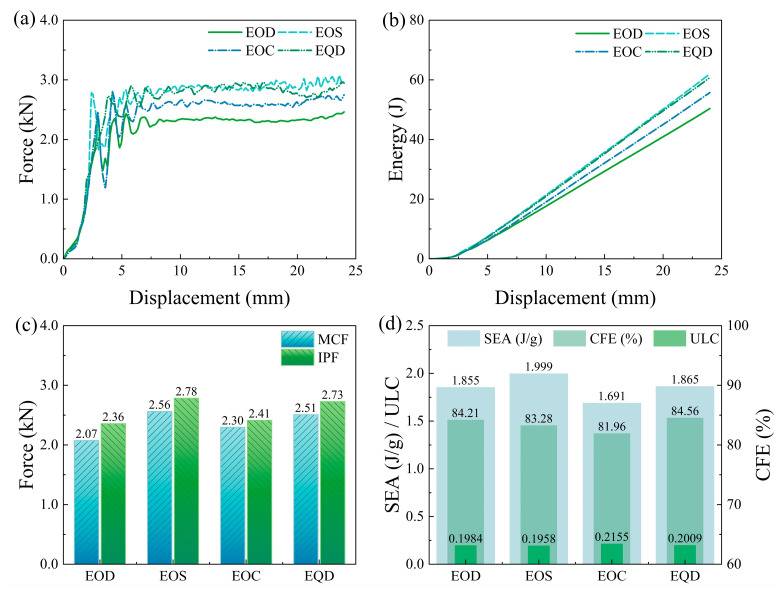
The crashworthiness indexes of the four types of combination structures (Group E): (**a**) force–displacement curves; (**b**) energy absorption curves; (**c**) IPF and MCF; (**d**) SEA, CFE, and ULC.

As depicted in Figure 9a, the load-carrying capacity of the combined structures is enhanced, with the curves not exhibiting significant peaks and valleys. The overall energy absorption effect is relatively smooth. In Figure 9b, the energy absorption-displacement curves of the four combined structures have essentially consistent slopes, further demonstrating the stability of the combined structures in energy absorption.

Figure 9c shows the MCF and IPF of the four combined structures. The smallest difference is in the EOC structure, with a difference of 4.7% between MCF and IPF, while the largest difference is in the EOD structure, with a difference of 14%, all below 15%.

Comparing Figure 7d and Figure 9d, it can be seen that the combined structures have varying degrees of improvement in overall performance. The EOS structure, combined with FOS and FQC, has a higher SEA than the FOS structure, with a 5.75% increase in CFE and only a 0.0037 increase in ULC compared to FOS. Relative to the FQC structure, the EOS structure has a 41.57% increase in SEA, a 16.81% increase in CFE, and a 0.0651 decrease in ULC.

Contrary to conventional wisdom, structures with high energy absorption performance can complement those with lower performance, forming combinations based on their distinct characteristics to enhance overall energy absorption.

### 4.3. Structural Optimization Analysis

Through the analysis in Section 4.2, it is evident that the designed bionic combined structure exhibits excellent energy absorption capability. During the optimization process, the distance *d* from the edge line to the curve vertex and the length-to-width ratio *y* (*y = c/b*) of the negative Poisson’s ratio structure beam were utilized. Except for the above two parameters, other dimensions remained constant in this section. In the parameter analysis, a full factorial experimental design method with 2 factors and 5 levels was employed to select the experimental sample points. Consequently, *d* was set to 4.4 mm, 4.6 mm, 4.8 mm, 5.0 mm, and 5.2 mm; *y* was set to 0.8, 0.9, 1.0, 1.1, and 1.2.

**Figure 10:**
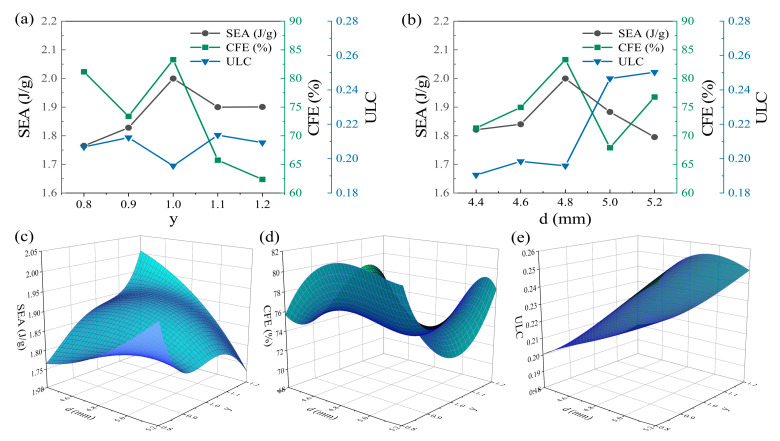
Influence of parameters on crash resistance index under compression condition: (**a**) *y*; (**b**) *d*. Spatial fitting surface established according to experimental data: (**c**) SEA; (**d**) CFE; (**e**) ULC.

Figure 10a illustrates the influence of different *d* and *y* values on the crashworthiness of the EOS structure under compression. As depicted in Figure 10a, an increase in y initially raises and subsequently lowers the SEA. For CFE, it initially decreases and then increases within the range of 0.8 to 1.0, followed by a gradual decline after reaching 1.0. The variation in y has minimal impact on ULC, since no significant increasing or decreasing trend is observed as y increases.

As revealed in Figure 10b, as d gradually increases, the SEA initially rises and then falls, while the CFE exhibits an overall fluctuating trend. The SEA attains its peak value at d = 4.8 mm, with SEA = 1.999 J/g and CFE = 83.28%. In contrast, the minimum value of ULC is maintained at d = 4.4 mm, with ULC = 0.1905. While the wall thickness progressively increases, the ULC demonstrates an overall upward trend, as shown in Table 3.

To optimize the EOS structure and achieve its optimal crashworthiness performance, the distance *d* from the edge line to the curve vertex and the length-to-width ratio *y* (*y* = *c/b*) of the negative Poisson’s ratio structure beam were employed as variables. The values for *d* were set at 4.4 mm, 4.6 mm, 4.8 mm, 5.0 mm, and 5.2 mm, while *y* was varied between 0.8, 0.9, 1.0, 1.1, and 1.2. To ensure the precision of the optimization meta-model, a full factorial experimental design was utilized, resulting in a total of 25 experimental trials. The experimental data are presented in Table 4. To maintain accuracy, a fifth-order polynomial was chosen to fit the models of SEA, CFE, and ULC based on the experimental data:

(7) $$\begin{array}{c} SEA(y,d)=a_{0}+a_{1}y+a_{2}d+a_{3}y^{2}+a_{4}yd+a_{5}d^{2}+a_{6}y^{3}\\ \;\;\;\;\;\;\;\;\;\;\;+a_{7}y^{2}d+a_{8}yd^{2}+a_{9}d^{3}+a_{10}y^{4}+a_{11}y^{3}d\\ \;\;\;\;\;\;\;\;\;\;\;\;\;\;\;\;\;+a_{12}y^{2}d^{2}+a_{13}yd^{3}+a_{14}d^{4}+a_{15}y^{5}+a_{16}y^{4}d\\ \;\;\;\;\;\;+a_{17}y^{3}d^{2}+a_{18}y^{2}d^{3}+a_{19}yd^{4}+a_{20}d^{5} \end{array}$$

(8) $$\begin{array}{c} CFE(y,d)=b_{0}+b_{1}y+b_{2}d+b_{3}y^{2}+b_{4}yd+b_{5}d^{2}+b_{6}y^{3}\\ \;\;\;\;\;\;\;\;\;\;\;+b_{7}y^{2}d+b_{8}yd^{2}+b_{9}d^{3}+b_{10}y^{4}+b_{11}y^{3}d\\ \;\;\;\;\;\;\;\;\;\;\;\;\;\;\;\;\;+b_{12}y^{2}d^{2}+b_{13}yd^{3}+b_{14}d^{4}+b_{15}y^{5}+b_{16}y^{4}d\\ \;\;\;\;\;\;+b_{17}y^{3}d^{2}+b_{18}y^{2}d^{3}+b_{19}yd^{4}+b_{20}d^{5} \end{array}$$

(9) $$\begin{array}{c} ULC(y,d)=c_{0}+c_{1}y+c_{2}d+c_{3}y^{2}+c_{4}yd+c_{5}d^{2}+c_{6}y^{3}\\ \;\;\;\;\;\;\;\;\;\;\;+c_{7}y^{2}d+c_{8}yd^{2}+c_{9}d^{3}+c_{10}y^{4}+c_{11}y^{3}d\\ \;\;\;\;\;\;\;\;\;\;\;\;\;\;\;\;\;+c_{12}y^{2}d^{2}+c_{13}yd^{3}+c_{14}d^{4}+c_{15}y^{5}+c_{16}y^{4}d\\ \;\;\;\;\;\;+c_{17}y^{3}d^{2}+c_{18}y^{2}d^{3}+c_{19}yd^{4}+c_{20}d^{5} \end{array}$$

The accuracy of the meta-model fitting is assessed using the Mean Relative Error (MRE) and Root Mean Square Error (RMSE) [40], with the corresponding calculation formulas given by

(10) $$MRE=\frac{1}{k}\sum_{i=1}^{k}\left|\frac{F_{i}(x)-F_{i}^{\prime }(x)}{F_{i}(x)}\right|\times 100\%$$

(11) $$RMSE=\sqrt{\frac{1}{k}\sum_{i=1}^{k}{\left(F_{i}(x)-F_{i}^{\prime }(x)\right)}^{2}}$$

In these formulas, *F_i_*(*x*) represents the *i*th crashworthiness simulation value, *F′_i_*(*x*) denotes the *i*th crashworthiness computed value, and *k* is the number of samples.

Figure 10c–e show the results of spatial surface fitting of SEA, CFE, and ULC, respectively. The results of the parameters of SEA, CEF and ULC are shown in Table 5.

Table 6 summarizes the model fitting error analysis. The MRE of SEA, CFE, and ULC are all within 3%, indicating that the established meta-model can predict the optimization target with high accuracy. The maximum values of SEA and CFE are obtained in the intervals of *y* (0.8,1.2) and *d* (4.4,5.2); the minimum value of ULC is obtained in the intervals of *y* (0.8,1.2) and *d* (4.4,5.2). Taking the maximum values of each index as the optimization target, the multi-objective optimization problem is solved using the genetic algorithm. Equation (12) is the optimization equation.

(12) $$\left\{\begin{array}{l} \max\left[SEA\left(d,y\right),CFE\left(d,y\right)\right]\\ \;\;\;\;\;\;\;\;\min\left[ULC(d,y)\right]\\ \;\;\;\;\;\;\;\;\;\;s.t.\left\{\begin{array}{l} 4.4\leq d\leq 5.2\\ 0.8\leq y\leq 1.2 \end{array}\right. \end{array}\right.$$

**Figure 11:**
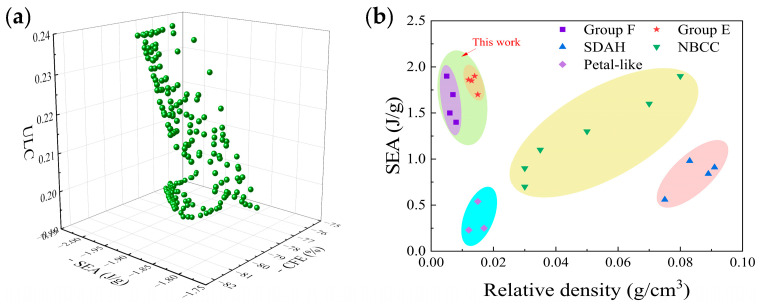
(**a**) The optimization model of the structure Pareto frontier; (**b**) comparative analysis of the Ashby diagram of SEA.

Figure 11a displays the Pareto front of the optimized model of the EOS structure. The optimal solutions are selected based on different needs. Considering the comprehensive structural performance, a set with relatively the best mechanical properties is chosen and verified through finite element model analysis. The verification results are shown in Table 7. The errors of the index data are all within 2.5%, and the predicted results meet the expectations. After optimization, the SEA, CFE, and ULC of the EOS structure are increased/decreased by 1.98%, 2.42%, and 2.05%, respectively.

Figure 11b compares the structures from this study with those from other research [41,42,43]. This comparison intuitively shows the advantages of the bionic Pistol Shrimp’s claw energy absorption structure designed in this study. The results indicate that under quasi-static compression and low-speed impact loads, the mechanical properties of the bionic energy absorption structure based on the Pistol Shrimp’s claw exceed those of traditional auxiliary structures and some novel 3D-printed structures to a certain extent.

## 5. Potential Applications of the Structures

**Figure 12:**
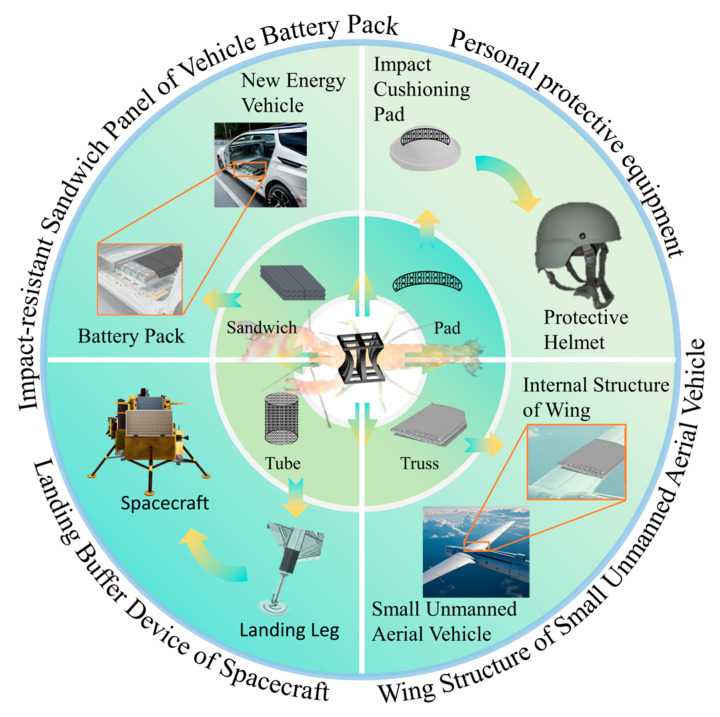
Potential application scenarios of the bionic Pistol Shrimp’s claw negative Poisson’s ratio structure.

Figure 12 illustrates several potential applications of the bionic Pistol Shrimp’s claw negative Poisson’s ratio structure as energy absorbers and cushioning devices. For instance, the upper right of Figure 12 describes a protection helmet, primarily used to protect the head during hazardous activities or while driving special vehicles. The lower right shows a support structure inside the wing of a small unmanned aerial vehicle, meeting its lightweight requirements. The lower left depicts the structure as a cushioning energy absorption component for spacecraft landing, which can be installed in multiple legs of the spacecraft to alleviate the impact during landing. The upper left is a protective cushioning layer for new energy vehicle batteries, which can protect the battery pack from accidental damage during vehicle operation and mitigate the energy impact on passengers in case of battery failure. However, this study only considered the mechanical response of the structure under low-speed impact loads. To further understand their structural performance, it is crucial to investigate their resistance to high-speed impact loads and bending properties. Finally, it is envisioned that the structure has potential applications in the fields of construction and aerospace engineering.

## 6. Conclusions

This study, in accordance with the current research trends in energy absorption structures and based on bionic design principles, draws inspiration from the Pistol Shrimp’s claw’s ability to withstand high-speed impacts generated by its own claw during predation without sustaining damage, to design bionic energy absorption structures. A biological characteristic curve was fitted from the cross-section of the Pistol Shrimp’s fixed claw through scanning electron microscopy experiments, and four types of bionic hierarchical porous metamaterial lattice structures with a negative Poisson’s ratio were designed based on its microstructure.

After validating the effectiveness of the finite element simulation model through quasi-static compression tests, the crashworthiness indicators of the four structures were tested and analyzed in a finite element model with a constant speed of 10 m/s. The results indicate that while the four structures may exhibit a significant advantage in a single evaluation index, they concurrently display notable shortcomings in other indicators.

To address this issue, four new combined bionic lattice structures were developed by integrating the characteristics of the original four structures, and their crashworthiness was analyzed using the same methodology. The four combined structures demonstrate superior balance across various indicators. The advantageous indicators were slightly enhanced or diminished, while the disadvantageous indicators were notably compensated. The crashworthiness of the EOS structure was evaluated to be superior. This demonstrates that the new structures obtained through coupling design can balance the structural performance and compensate for the deficiencies of individual structures, representing a viable design approach for material lattice design.

To further enhance the EOS structure, this study performed 25 simulations for two parameters: the distance d from the edge line to the curve vertex, and the length-to-width ratio *y* (*y* = *c*/*b*) of the negative Poisson’s ratio structure beam. A five-order polynomial was employed to fit the three evaluation indicators of the EOS structure, namely SEA, CFE, and ULC, to create a three-dimensional surface prediction model. A genetic algorithm was utilized to optimize the three indicators, resulting in an increase/decrease of 1.98%, 2.42%, and 2.05% in the SEA, CFE, and ULC of the EOS structure, respectively. The EOS structure, after coupling design, can be further optimized through a genetic algorithm. This indicates that coupling design can be combined with other optimization methods to provide a new approach for subsequent research on material lattice structures with superior performance.

Finally, the potential application fields of the bionic hierarchical porous metamaterial lattice structure were illustrated with a variety of examples.

## Figures and Tables

**Table 1:** Parameters of the model.

Specimen	h (mm)	a (mm)	b (mm)	c (mm)	d (mm)
Value	20	20	2	2	4.8

**Table 2:** Comparison of simulation and experimental data.

	SEA (J/g)	PF (kN)	CFE (%)	EA (J)	m (g)
Experiment 1	1.012	2.815	67.25	32.79	32.40
Experiment 2	1.050	2.741	62.04	34.02	32.41
Simulation	1.031	2.685	65.05	33.28	33.27
Errors 1	1.877%	−4.618%	−3.271%	1.488%	2.685%
Errors 2	−1.809%	−2.043%	4.851%	−2.175%	2.653%

**Table 3:** Range crashworthiness indexes under compression conditions.

	SEA (J/g)		CFE (%)		ULC	
	*d* (mm)	*y*	*d* (mm)	*y*	*d* (mm)	*y*
Max	1.999	1.999	83.28	83.28	0.2503	0.2136
Min	1.795	1.764	67.90	62.37	0.1905	0.1958
Range	0.204	0.235	15.38	20.91	0.0598	0.0178

**Table 4:** Crashworthiness of EOS structures with different combinations of parameters.

Number	Test Parameters	*y*	*d* (mm)	SEA (J/g)	CFE (%)	ULC
1	EOS-0844	0.8	4.4	1.791	68.28	0.2068
2	EOS-0846	0.8	4.6	1.778	82.79	0.1983
3	EOS-0848	0.8	4.8	1.764	81.16	0.2068
4	EOS-0850	0.8	5.0	1.928	81.91	0.2448
5	EOS-0852	0.8	5.2	1.968	79.72	0.2384
6	EOS-0944	0.9	4.4	1.886	75.28	0.2074
7	EOS-0946	0.9	4.6	1.849	84.25	0.1992
8	EOS-0948	0.9	4.8	1.827	73.38	0.2122
9	EOS-0950	0.9	5.0	1.862	70.69	0.2445
10	EOS-0952	0.9	5.2	1.812	76.92	0.2491
11	EOS-1044	1.0	4.4	1.821	71.33	0.1905
12	EOS-1046	1.0	4.6	1.840	74.95	0.1983
13	EOS-1048	1.0	4.8	1.999	83.28	0.1958
14	EOS-1050	1.0	5.0	1.883	67.90	0.2466
15	EOS-1052	1.0	5.2	1.795	76.76	0.2503
16	EOS-1144	1.1	4.4	1.940	78.49	0.2104
17	EOS-1146	1.1	4.6	1.904	81.52	0.1984
18	EOS-1148	1.1	4.8	1.899	65.73	0.2136
19	EOS-1150	1.1	5.0	1.916	74.89	0.2323
20	EOS-1152	1.1	5.2	1.782	78.15	0.2404
21	EOS-1244	1.2	4.4	2.007	76.56	0.1897
22	EOS-1246	1.2	4.6	1.983	75.89	0.1943
23	EOS-1248	1.2	4.8	1.900	62.37	0.2093
24	EOS-1250	1.2	5.0	1.853	71.39	0.2240
25	EOS-1252	1.2	5.2	1.756	79.84	0.2230

**Table 5:** Parameter results of fitted equations.

SEA		CFE		ULC	
a_0_	2.9605	b_0_	−86.546	c_0_	1.7241
a_1_	−2.405	b_1_	17.802	c_1_	−0.83948
a_2_	0.7367	b_2_	−166.71	c_2_	−0.14938
a_3_	−2.9261	b_3_	5.4971	c_3_	2.3017
a_4_	−3.1322	b_4_	−0.17192	c_4_	−0.76637
a_5_	0.46923	b_5_	−117.94	c_5_	−0.17326
a_6_	−6.2113	b_6_	55.746	c_6_	0.07251
a_7_	−5.9668	b_7_	31.233	c_7_	−1.8144
a_8_	6.0231	b_8_	93.305	c_8_	0.84037
a_9_	−0.99341	b_9_	112.16	c_9_	−0.01113
a_10_	2.607	b_10_	−9.9687	c_10_	−2.8341
a_11_	−2.199	b_11_	28.834	c_11_	2.5997
a_12_	5.2731	b_12_	36.477	c_12_	−0.42608
a_13_	−2.4319	b_13_	−66.711	c_13_	−0.09048
a_14_	0.33693	b_14_	−20.733	c_14_	0.01065
a_15_	12.963	b_15_	−0.20484	c_15_	0.89787
a_16_	−5.5242	b_16_	−28.327	c_16_	−0.39991
a_17_	−0.93977	b_17_	−13.259	c_17_	−0.09297
a_18_	0.33361	b_18_	2.2156	c_18_	0.04023
a_19_	0.07617	b_19_	6.8682	c_19_	0.00372
a_20_	−0.02146	b_20_	1.1234	c_20_	−0.0011

**Table 6:** Model error analysis.

Index	MRE	RMSE
SEA	1.0437%	0.0250 J/g
CFE	2.9459%	2.8589%
ULC	2.3433%	0.0063

**Table 7:** Validation results.

	*y*	*d* (mm)	SEA (J/g)	CFE (%)	ULC
Emulation	1.1868	4.408	2.02754	79.7521	0.18972
Optimal Solution			1.9872	77.8142	0.1936
RE (%)	-	-	1.98%	2.42%	2.05%

## Data Availability

Other researchers can access the data supporting the conclusions of the study. (1) The nature of the data is the source data of the image in the paper; (2) Some or all of the data that support the findings of this study are available from the author by sending an email to w2558123741@163.com or iansongjiafeng@163.com upon request; (3) There is no restrictions on the data access.

## References

[1] Wen G., Zhang S., Wang H., Wang Z.-P., He J., Chen Z., Liu J., Xie Y.M. (2023). Origami-based acoustic metamaterial for tunable and broadband sound attenuation. Int. J. Mech. Sci..

[2] Wang X., Li Z., Deng J., Gao T., Zeng K., Guo X., Li X., Zhai W., Wang Z. (2025). Unprecedented Strength Enhancement Observed in Interpenetrating Phase Composites of Aperiodic Lattice Metamaterials. Adv. Funct. Mater..

[3] Li Q., Zhan L., Miao X., Hu L., Li E., Zou T. (2022). Morning glory-inspired lattice structure with negative Poisson’s ratio effect. Int. J. Mech. Sci..

[4] Liu H.T., Liu J.Y., Wu W.J. (2024). Energy absorption characteristics and multi-objective optimization of 3D bionic negative Poisson’s ratio honeycomb. Mater. Today Commun..

[5] Chen S., Lian X., Liu X., Hu J., Wang B., Xu J., Wu L. (2025). Negative stiffness mechanical metamaterials based on curved beams for reusable shock isolation. Int. J. Smart Nano Mater..

[6] Li Z., Li X., Guo Z., Zhou Y., Lin J., Mo Z., Li J. (2024). Mechanical response of a novel square dome shell with bistable behavior: Improved analytical method and empirical model. Thin-Walled Struct..

[7] Nicolaou Z.G., Jiang F., Motter A.E. (2024). Metamaterials with negative compressibility highlight evolving interpretations and opportunities. Nat. Commun..

[8] Wang Q., Li Z., Zhang Y., Cui S., Yang Z., Lu Z. (2020). Ultra-low density architectured metamaterial with superior mechanical properties and energy absorption capability. Compos. Part B Eng..

[9] Zheng X., Xiao Z., Ren Z., Zi B., Wu Y., Yao L., Bai H. (2024). Entangled metallic porous material–silicone rubber interpenetrating phase composites with simultaneous high specific stiffness and energy consumption. Compos. Struct..

[10] Hsieh M.-T., Endo B., Zhang Y., Bauer J., Valdevit L. (2019). The mechanical response of cellular materials with spinodal topologies. J. Mech. Phys. Solids.

[11] Dong Z., Li Y., Zhao T., Wu W., Xiao D., Liang J. (2019). Experimental and numerical studies on the compressive mechanical properties of the metallic auxetic re-entrant honeycomb. Mater. Des..

[12] Liu J.Y., Liu H.T. (2022). Energy absorption characteristics and stability of novel bionic negative Poisson’s ratio honeycomb under oblique compression. Eng. Struct..

[13] Wu H.X., Zhang X.C., Liu Y. (2020). In-plane crushing behavior of density graded cross-circular honeycombs with zero Poisson’s ratio. Thin-Walled Struct..

[14] Nian Y., Wan S., Avcar M., Wang X., Hong R., Yue R., Li M. (2024). Nature-inspired 3D printing-based double-graded aerospace negative Poisson’s ratio metastructure: Design, fabrication, investigation, optimization. Compos. Struct..

[15] Song Z., Liang H., Ding H., Ma M. (2023). Structure design and mechanical properties of a novel anti-collision system with negative Poisson’s ratio core. Int. J. Mech. Sci..

[16] Xinyi L., Yifeng Z., Rong L., Yilin Z., Evrard I.A. (2024). Static and modal analysis of sandwich panels with rib-reinforced re-entrant honeycomb. Int. J. Mech. Sci..

[17] Xiao R., Feng X., Liu W., Zhou W., Li X., Song I., Ding M., Pu Y., Zhang D., Fan R. (2023). Direct 3D printing of thin-walled cardiovascular stents with negative Poisson’s ratio structure and functional metallic coating. Compos. Struct..

[18] Ren J.P., Gu Z.P., Zhao A.G., Huang C.G., Wu X.Q. (2025). Enhancing energy absorption of star-shaped honeycombs by utilizing negative Poisson’s ratio effect under high-velocity impact. Int. J. Impact Eng..

[19] Qi C., Jiang F., Yu C., Yang S. (2019). In-plane crushing response of tetra-chiral honeycombs. Int. J. Impact Eng..

[20] Zeng W., Jiang W., Liu J., Huang W. (2022). Fabrication method and dynamic responses of composite sandwich structure with re-entrant honeycomb cores. Compos. Struct..

[21] Jin Y.-T., Qie Y.-H., Li N.-N., Li N.-W. (2022). Study on elastic mechanical properties of novel 2D negative Poisson’s ratio structure: Re-entrant hexagon nested with star-shaped structure. Compos. Struct..

[22] Wang S., Liu H.T., Cai G.B. (2024). Programmable mechanical responses of a hybrid star-rhombus honeycomb based on digital design method. Thin-Walled Struct..

[23] Zhang W., Wang H., Lou X., Yan Z., Shao J., Wu T., Qin Q. (2024). On in-plane crushing behavior of a combined re-entrant double-arrow honeycomb. Thin-Walled Struct..

[24] Wang H., Lu Z., Yang Z., Li X. (2019). In-plane dynamic crushing behaviors of a novel auxetic honeycomb with two plateau stress regions. Int. J. Mech. Sci..

[25] Yu S., Liu Z., Cao X., Liu J., Huang W., Wang Y. (2023). The compressive responses and failure behaviors of composite graded auxetic re-entrant honeycomb structure. Thin-Walled Struct..

[26] Wu Y., Sun L., Yang P., Fang J., Li W. (2021). Energy absorption of additively manufactured functionally bi-graded thickness honeycombs subjected to axial loads. Thin-Walled Struct..

[27] Wang T., An J., He H., Wen X., Xi X. (2021). A novel three-dimensional impact energy absorption structure with negative Poisson’s ratio and its application in aircraft crashworthiness. Compos. Struct..

[28] Santos F., Rebelo H., Coutinho M., Sutherland L., Cismasiu C., Farina I., Fraternali F. (2021). Low velocity impact response of three-dimensional printed structures formed by cellular metamaterials and stiffening plates: PLA vs. PETg. Compos. Struct..

[29] Liu R., Yao G., Xu Z., Yu Z., Zhang Z., Han C., Li H., Jiang S. (2022). Study on quasi-static mechanical properties of novel re-entrant structures with multiple energy dissipation. Thin-Walled Struct..

[30] Wang P., Yang F., Lu G., Bian Y., Zhang S., Zheng B., Fan H. (2022). Anisotropic compression behaviors of bio-inspired modified body-centered cubic lattices validated by additive manufacturing. Compos. Part B Eng..

[31] Wang X., Li X., Li Z., Wang Z., Zhai W. (2024). Superior Strength, Toughness, and Damage-Tolerance Observed in Microlattices of Aperiodic Unit Cells. Small.

[32] Lazarus B.S., Velasco-Hogan A., Gómez-del Río T., Meyers M.A., Jasiuk I. (2020). A review of impact resistant biological and bioinspired materials and structures. J. Mater. Res. Technol..

[33] Abdullah N.A.Z., Sani M.S.M., Salwani M.S., Husain N. (2020). A review on crashworthiness studies of crash box structure. Thin-Walled Struct..

[34] Sun Z., Gong Y., Bian Z., Zhang J., Zhao L., Hu N. (2024). Mechanical properties of bionic lattice and its hybrid structures based on the microstructural design of pomelo peel. Thin-Walled Struct..

[35] Cui C.Y., Chen L., Feng S., Cui X.G., Lu J.Z. (2023). Novel cuttlebone-inspired hierarchical bionic structure enabled high energy absorption. Thin-Walled Struct..

[36] Wei W., Quan X., Cao H., Zhang S., Zhao X., Yu N., Zhou J., Wang H., Hou X. (2021). Research on the rapid closing jet mechanism of pistol shrimp’s claws based on fluid dynamic grid. Math. Probl. Eng..

[37] Knight K.C. (2023). Even the tiniest snapping shrimp claws make cracking pistol pops. J. Exp. Biol..

[38] Li X., Li Z., Guo Z., Mo Z., Li J. (2023). A novel star-shaped honeycomb with enhanced energy absorption. Compos. Struct..

[39] Liang H.Y., Sun B.H., Hao W.Q., Sun H., Pu Y.F., Ma F.W. (2022). Crashworthiness of lantern-like lattice structures with a bidirectional gradient distribution. Int. J. Mech. Sci..

[40] Alizamir M., Kim S., Ikram R.M.A., Ahmed K.O., Heddam S., Gholampour A. (2025). A reliable hybrid extreme learning machine–metaheuristic framework for enhanced strength prediction of 3D-printed fiber-reinforced concrete. Results Eng..

[41] Li X., Li Z., Guo Z., Guo Z., Mo Z., Li J. (2025). A novel hybrid star honeycomb with individually adjustable second plateau stresses. Compos. Struct..

[42] Ma X., Zhang N., Zhang C., Tian X. (2024). Mechanical behavior of a novel lattice structure with two-step deformation. Thin-Walled Struct..

[43] Li Z.-Y., Zhang W.-M., Wang W.-J., Ho M.M.P., Xiong J., Yang J.-S., Wang X.-T., Zhang M., Hu H. (2024). New 3D petal-like structures with lightweight, high strength, high energy absorption, and auxetic characteristics. Thin-Walled Struct..

